# Extract of *Ganoderma sinensis* spores induces cell cycle arrest of hepatoma cell via endoplasmic reticulum stress

**DOI:** 10.1080/13880209.2021.1931354

**Published:** 2021-06-10

**Authors:** Weiming Lin, Li Gu, Ling-Yan Zhu, Sha Zhou, Danhong Lian, Yongquan Xu, Limin Zheng, Xin Liu, Lian Li

**Affiliations:** a MOE Key Laboratory of Gene Function and Regulation, School of Life Sciences, Sun Yat-sen University, Guangzhou, China; b Academy of Food and Health Engineering, Sun Yat-Sen University, Guangzhou, China

**Keywords:** Antitumor activity, hepatocellular carcinoma, unfolded protein response

## Abstract

**Context:**

*Ganoderma sinensis* Zhao, Xu et Zhang (Ganodermataceae) has been used for the prevention or treatment of a variety of diseases, including cancer.

**Objective:**

We investigated the antitumor activity and mechanism of an extract from *G. sinensis* against hepatocellular carcinoma.

**Materials and methods:**

A *G. sinensis* extract (GSE) was obtained from sporoderm-broken *G. sinensis* spores by supercritical fluid carbon dioxide extraction. Hepatoma cells, HepG2 cells, were treated with emulsified sample of GSE at 12.5, 25, 50, 100 and 150 μg/mL for 24 h. The Alamar Blue assay was used to examine growth inhibitory effects. Changes in cell structure and morphology were assessed via transmission electron microscopy and confocal laser scanning microscope. Cell cycle distribution was analysed by flow cytometry.

**Results:**

GSE suppressed the proliferation of HepG2 cells (IC_50_=70.14 μg/mL). Extensive cytoplasmic vacuolation originating from dilation of the endoplasmic reticulum (ER) was shown in GSE-treated HepG2 cells. GSE treatment also upregulated the expression of ER stress-related proteins in HepG2 cells. Cells tended to be arrested at the G2/M cell cycle stage after GSE treatment (30.8 ± 1.4% and 42.2 ± 2.6% at GSE with 50 μg/mL and 100 μg/mL vs. 21.03 ± 1.10%, control). Pre-treatment with salubrinal, an inhibitor of ER stress, effectively attenuated cell cycle arrest induced by GSE.

**Discussion and conclusions:**

Our findings provide new evidence that GSE suppresses growth of cancer cells *in vitro* through activating the ER stress pathway. The GSE may be clinically applied in the prevention and/or treatment of cancer.

## Introduction

Hepatocellular carcinoma (HCC), the most frequent primary malignancy of the liver, has become the fourth leading cause of cancer-associated death worldwide (Bray et al. 2018). At present, surgical resection, orthotopic liver transplantation and radiofrequency thermal ablation are the most common treatments for HCC. Although therapeutic advances have recently been made, the prognosis of HCC remains unsatisfactory, due to resistance to routine chemotherapeutic agents, high recurrence and metastasis. Therefore, novel anticancer drugs with increased selectivity for cancer cells and reduced systemic toxicity are required to improve HCC outcomes.

*Ganoderma sinensis* Zhao, Xu et Zhan (Ganodermataceae) is an herbal medicine that has been used for thousands of years to prevent or treat a variety of diseases, including cancer. Triterpenoids and polysaccharides are regarded as the major active anticancer components extracted from *G. sinensis*. Generally, polysaccharides serve as immunomodulators or antioxidants to combat cancer, while triterpenoids mainly inhibit cancer cell proliferation and metastasis (Wu et al. 2013). We have obtained an extract from *G. sinensis* spores with a high bioactive triterpenoid content by supercritical fluid carbon dioxide extraction, which exerts direct tumoricidal effects (Zhang et al. 2009); however, its underlying mechanism of action is unclear.

The endoplasmic reticulum (ER) is responsible for protein folding and protein quality control, lipid synthesis and calcium storage. Pathological, environmental or physiological stimuli can cause ER stress, which in turn, affect various biological processes, such as proliferation, metabolism, inflammation, autophagy and apoptosis (Kim et al. 2008). ER stress responses are mediated by the activation of several unfolded protein response (UPR) signalling pathways, which are predominantly controlled by three major sensors: inositol requiring enzyme 1 (IRE1), protein kinase RNA-activated (PKR)-like ER kinase (PERK) and activating transcription factor 6 (ATF6) (Maurel et al. 2015). Activation of the UPR can increase the overall protein folding capacity and reduce the protein load of the ER.

Some naturally occurring compounds have been found to decrease proliferation and increase apoptosis in various cancer cells by activating the ER stress response and the UPR pathways (Liu et al. 2016; Ham et al. 2018; Ma et al. 2018); however, whether compounds in the *G. sinensis* extract (GSE) work via a similar mechanism remains unclear. In the present study, we investigate the effect and mechanism of the extract of *G. sinensis* spores on the survival and proliferation of an HCC cell line.

## Materials and methods

### Reagents

Foetal bovine serum (FBS) and Dulbecco's modified Eagle's medium (DMEM) were obtained from Thermo Fisher Scientific (Waltham, MA) and 4′,6-diamidino-2-phenylindole (DAPI) was from Sigma-Aldrich (Seelze, Germany). ER-tracker Red was obtained from Thermo Fisher Scientific (Waltham, MA). Mouse monoclonal antibodies (mAbs) against PERK, phosphorylated-eIF2a, CHOP, XBP-1s, cyclin A2 and Cdc25C were obtained from Cell Signaling Technology (Danvers, MA); and anti-β-actin and CDK1 were from Boster (Pleasanton, CA). Salubrinal was purchased from Calbiochem (San Diego, CA).

### Plant and spores

A specimen of *G. sinensis* was obtained and deposited in the Mycological Herbarium, Institute of Microbiology, Chinese Academy of Sciences (Lian et al. 2019). The high-quality *G. sinensis* spores were collected in August 2017, as described in our previous work (Li et al. 2016). They were identified by Dr. Wenhua He, Institute of Microbiology, Chinese Academy of Sciences.

### Sample preparation and determination of ganoderic acids content

A GSE (27.5 g) was obtained from 100 g of sporoderm-broken *G. sinensis* spores, as described previously (yield = 27.5 g/100 g) (Li et al. 2016). The extract consisted of 85% triglycerides, 2% ergosterol and 10.88% triterpenoids. The triglyceride in the extract contains 18 fatty acids with the majority of C16:0, C16:1, C18:0, C18:1 and C18:2 (Liu et al. 2007), and ergosterol includes free ergosterol and ergosterol esters (Yuan et al. 2006). Total triterpenoid content in the extract was determined by measuring the absorbance at 548.1 nm using thin-layer chromatography–spectrophotometry and with ursolic acid as the standard. The result showed that the total triterpenoid content was 10.88%. Dressings were prepared by dissolving phosphatidylcholine (Central Soya Co., St. Louis, MO) in distilled water, and the solution was pre-emulsified in a blender for 1 min. Then, GSE was emulsified as previously described (Zhang et al. 2009). The emulsified sample contains 15.38% (w/w) of the extract of *G. sinensis* spores.

Ganoderic acids are the major bioactive triterpenoid component in *G. sinensis* and are considered ‘markers’ for chemical evaluation. The ganoderic acid content in the extract was determined as described previously (Gao et al. 2004; Li et al. 2016). Briefly, 20 g of the extract of *G. sinensis* spores was suspended in 75 mL of petroleum ether and partitioned twice with 75 mL of 95% MeOH. After *in vacuo* evaporation, the residue was suspended in 50 mL of dichloromethane and partitioned twice with 50 mL of water. The resulting dichloromethane soluble extract was dissolved in 5 mL of methanol and filtered through a 0.45 μM membrane. Then, 10 μL of the solution was injected into the HPLC System (Binary HPLC Pump 1525, Photodiode Array Detector 2996; Waters, Milford, MA), which included a ZORBAX SB-C18 column (4.6 mm × 250 mm, 5 μm; Agilent, Santa Clara, CA) and a Guard column (3.9 mm × 20 mm × 5 mm; Agilent, Santa Clara, CA). The mobile phase was made by mixing solvent A (0.1% phosphoric acid aqueous solution) and solvent B (acetonitrile) using the following gradient program: 0 min, 30% B; 10 min, 36% B; 40 min, 37% B; 50 min, 38% B. The flow rate was set at 1.0 mL/min, and the detecting wavelength was set at 254 nm. The operating temperature was maintained at 23 °C. The chromatographic peak was identified by comparing the retention times and spectra against known standards (Chan et al. 2017). The method was validated for parameters such as linearity, precision, repeatability and stability, as previously described (Gao et al. 2004).

### Cell culture and treatment

The human HCC cell line, HepG2, was originally obtained from the American Type Culture Collection (Manassas, VA). The normal liver epithelial cell line, HL-7702, was purchased from the Cell Bank of the Type Culture Collection (Chinese Academy of Sciences, Shanghai, China). The cells were cultured in DMEM medium supplemented with 10% FBS, 100 U/mL penicillin and 100 μg/mL streptomycin (Hyclone, Logan, UT) in a humidified cell incubator with an atmosphere of 5% CO_2_ at 37 °C. Cells were treated with GSE at various concentrations for the indicated periods.

### Cell viability assay

Cells were seeded at a density of 5 × 10^3^ cells/well in 96-well plates and allowed to attach overnight. The next day, they were treated with GSE or with medium alone (control) for the indicated time. The dressing solution was used as vehicle control. The viability of cells was determined by the Alamar Blue assay (AbD Serotec, Oxford, UK). The cell viability was calculated according to the following formula: inhibition rate (%)=(1 – absorbance of experimental group/absorbance of control group)×100%. Values are represented as means ± SEM of three independent experiments performed in triplicate.

### Wright–Giemsa staining

After treating the cells in the 12-well plate with GSE or vehicle, adherent and non-adherent cells were collected and washed with PBS. Cells were mounted on glass slides by Cytospin (CytoPro 7620, Wescor, Provo, UT), and morphological evaluation was assessed by Wright–Giemsa staining. Samples were dried overnight at room temperature and observed using a microscope (DMI 4000B, Leica, Wetzlar, Germany).

### Western blotting

Proteins were extracted as previously described and separated by 10% sodium dodecyl sulphate-polyacrylamide gel electrophoresis (SDS-PAGE) (Pan et al. 2019), then being immunoblotted with a mAb against ER stress or cell cycle-related molecules, and visualized with an ECL kit (Pierce, Rockford, IL); β-actin was used as an internal control.

### Cell cycle analysis

Flow cytometric analysis was performed using PI/RNase staining according to standard procedures. Cells were collected and then fixed with 75% ice-cold ethanol. After centrifugation, the cells were washed twice with ice-cold PBS, then stained with propidium iodide (PI) at 4 °C for 2 h in the dark. Cell cycle analysis was performed using a Gallios flow cytometer (Beckman Coulter, Brea, CA) and the FlowJo 10.0 software (Tree Star, San Carlos, CA).

### Apoptosis assay

HepG2 cells were treated with GSE or with the vehicle for 24 or 48 h. Adherent and non-adherent cells were collected and resuspended in Annexin V-binding buffer. Cells were stained with Annexin V-FITC and DAPI (eBioscience, San Diego, CA) for flow cytometric measurement of apoptosis. Data were acquired using a Cytoflex S flow cytometer (Beckman Coulter, Brea, CA) and analysed using FlowJo (Tree Star, San Carlos, CA).

### Transmission electron microscopy

Transmission electron microscopy (TEM) was used to observe the ER in HepG2 cells, as described previously (Dou et al. 2018). In brief, cells were fixed overnight at 4 °C with ice-cold 2.5% glutaraldehyde in 0.1 M PBS, and then fixed with 1% OsO_4_ for 1.5 h. After being washed and dehydrated with graded acetone, the samples were infiltrated and embedded in SPI-812 resin/Spurr resin. Ultrathin sections (80 nm) were cut, stained with uranyl acetate and lead citrate, and then examined with a transmission electron microscope (JEM 1400 JOEL, Tokyo, Japan).

### ER localization with ER-tracker staining

ER staining was performed using the ER-tracker kit according to the manufacturer’s instructions (Invitrogen, Carlsbad, CA). Briefly, cells were washed with PBS and then incubated in pre-warmed ER-tracker dye solution (1 mM) for approximately 30 min at 37 °C. After washing with PBS, the cells were observed using an Olympus confocal laser scanning microscope (Tokyo, Japan).

### Statistical analysis

All experiments were performed as three independent experiments (*n* = 3). Data are expressed as mean ± SEM. All statistical analyses were conducted using GraphPad Prism version 6.0 (GraphPad, San Diego, CA). Statistical differences between means were evaluated using a one-way analysis of variance (ANOVA) followed by Tukey’s pairwise comparisons. A *p* value <0.05 was considered statistically significant.

## Results

### Ganoderic acid content of the *G. sinensis* extract

**Figure 1.**
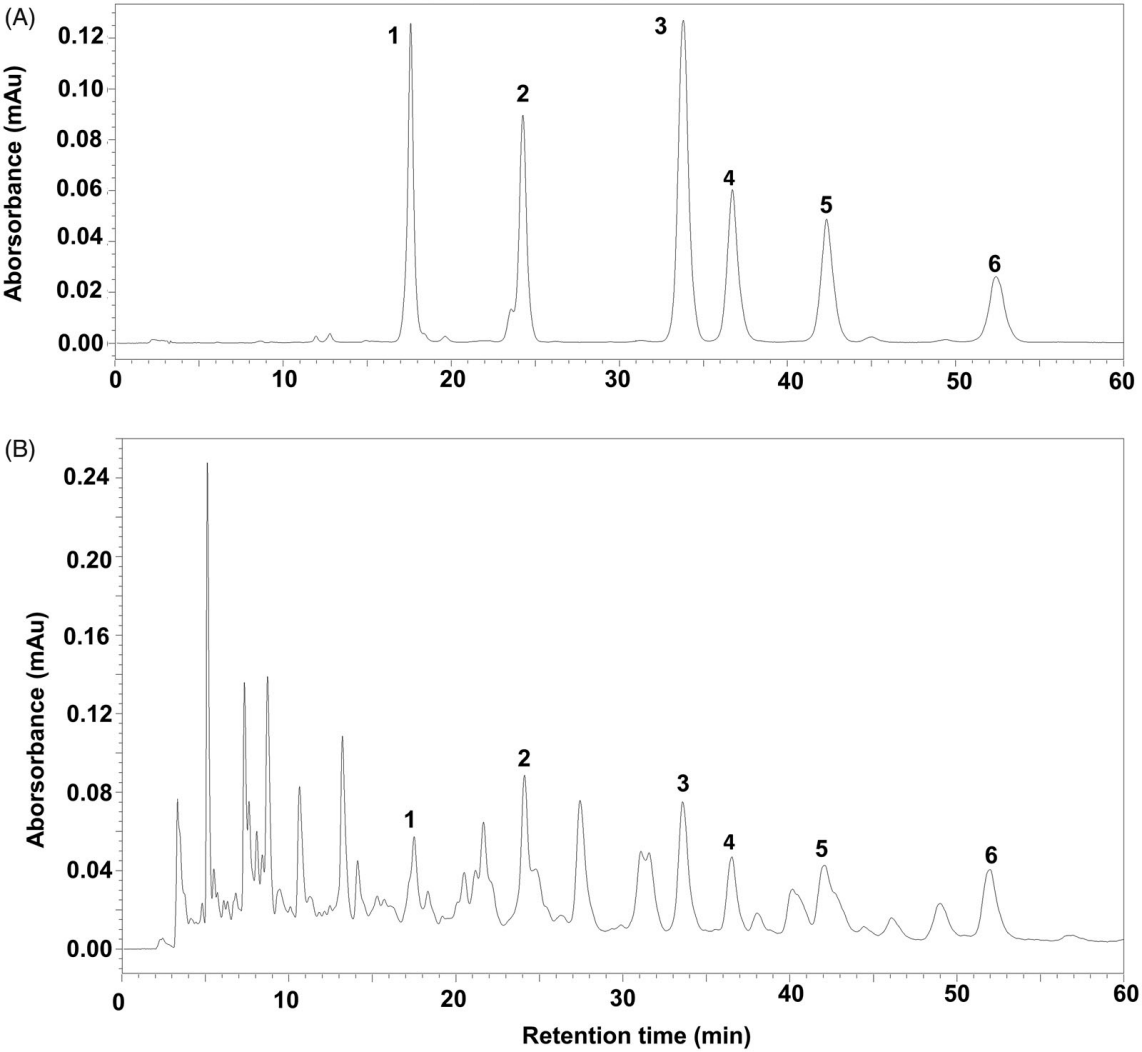
The HPLC chromatographic profile of ganoderic acids in the extract of *G. sinensis*. (A) Reference substance. (B) Sample of the extract of *G. sinensis*. (1) Ganoderenic acid B; (2) ganoderic acid A; (3) ganoderenic acid D; (4) ganoderic acid D; (5) lucidenic acid D; (6) ganolucidate F.

Ganoderic acid content of the extract was determined by HPLC. The method was validated in terms of linearity, precision and accuracy (Tables 1 and 2). Six ganoderic acids were identified and quantified from the HPLC chromatogram (Figure 1), including ganoderenic acid B (10.44 ± 0.20 μg/g), ganoderic acid A (21.60 ± 0.41 μg/g), ganoderenic acid D (8.67 ± 0.55 μg/g), ganoderic acid D (9.18 ± 0.80 μg/g), lucidenic acid D (13.04 ± 0.65 μg/g) and ganolucidate F (21.47 ± 0.81 μg/g).

**Table 1.** Retention time reproducibility, regression equations, correlation coefficients (*R*^2^) and linearity ranges of constituents of the extract of *G. sinensis*.

Analyte	Regression equation	*R* ^2^	Retention time (min, *n* = 5)	Linear range (μg/mL)
Ganoderenic acid B	*y* = 20707.8*x* – 13,927	0.9997	17.593 ± 0.066	49.10–982
Ganoderic acid A	*y* = 20062.0*x* – 9029.2	0.9996	24.265 ± 0.08	48.45–969
Ganoderenic acid D	*y* = 36903.6*x* – 30,517	0.9995	33.805 ± 0.12	48.95–979
Ganoderic acid D	*y* = 17883.6*x* – 12,345	0.9995	36.727 ± 0.121	48.9–978
Lucidenic acid D	*y* = 15647.4*x* – 11,184	0.9998	42.325 ± 0.272	49.25–985
Ganolucidate F	*y* = 10623.6*x* – 3244	0.9997	52.420 ± 0.104	49.15–983

**Table 2.** Precision, reproducibility, stability and recovery of constituents of the extract of *G. sinensis* (*n* = 6).

Analytes	Precision (% RSD)	Repeatability (% RSD)	Stability (% RSD)	Recovery (%)	Recovery (% RSD)
Ganoderenic acid B	0.32	0.65	1.83	93.26 ± 2.07	2.64
Ganoderic acid A	1.87	1.37	0.69	102.83 ± 2.12	2.37
Ganoderenic acid D	1.08	2.72	1.69	96.19 ± 0.97	1.49
Ganoderic acid D	1.47	1.12	1.49	91.45 ± 2.16	2.18
Lucidenic acid D	0.85	2.28	2.98	99.78 ± 3.04	3.02
Ganolucidate F	0.63	2.65	1.00	101.57 ± 1.83	1.79

%RSD: relative standard deviation, as a percentage.

### GSE inhibits growth and alters the morphology of HepG2 cells

**Figure 2.**
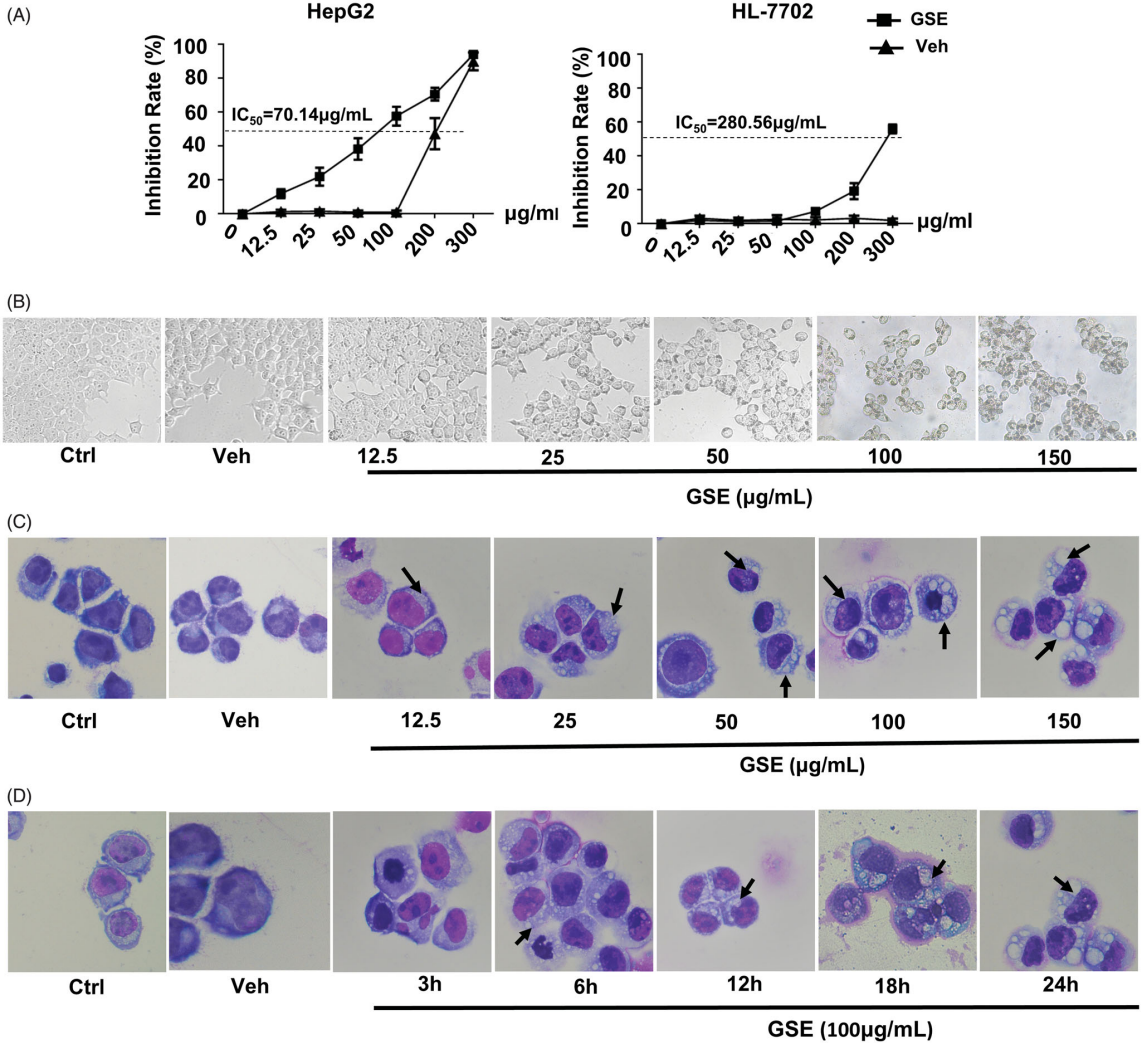
GSE treatment results in decreased cell growth and extensive cytoplasmic vacuolation in HepG2 cells. (A) The inhibitory effects of GSE on cell growth in HepG2 cells or normal liver epithelial cells HL-7702. Cells were treated with the indicated concentrations of GSE for 24 h, cumulative viable cells were measured by the Alamar Blue assay. Data are presented as the means ± SEM of three independent experiments. (B) Morphological alterations of HepG2 cells after treatment with GSE for 24 h by light microscopy (original magnification, ×200). (C) Morphological alterations of HepG2 cells after treatment with GSE by Wright–Giemsa staining. For dose–response experiments (C), cells were treated with GSE for 24 h. For time-course experiments, cells were treated with 100 μg/mL of GSE (D). Ctrl: control; Veh: vehicle.

The growth of HepG2 cells was significantly inhibited by GSE. At a concentration of 100 μg/mL, GSE suppressed HepG2 cell growth by about 60% after 24 h of treatment (IC_50_=70.14 μg/mL). In contrast, GSE at 100 μg/mL only showed a slight inhibitory effect on normal liver epithelial cells (HL-7702 cells; IC_50_=280.56 μg/mL (Figure 2(A)). When examining morphological changes via light microscopy, we observed extensive cytoplasmic vacuolation in HepG2 cells after treated with GSE (Figure 2(B,C)). In terms of vacuolization surrounding the cell nucleus, small vacuoles appeared in HepG2 cells at 6 h after GSE treatment. These small vacuoles gradually fused into giant vacuoles with increased exposure time (Figure 2(D)). After 24 h of GSE treatment, some HepG2 cells became detached from the culture plate. Together, these results indicate GSE selectively inhibits the growth of HepG2 cells and induces morphological changes in HCC.

### GSE causes dilation of the ER

**Figure 3.**
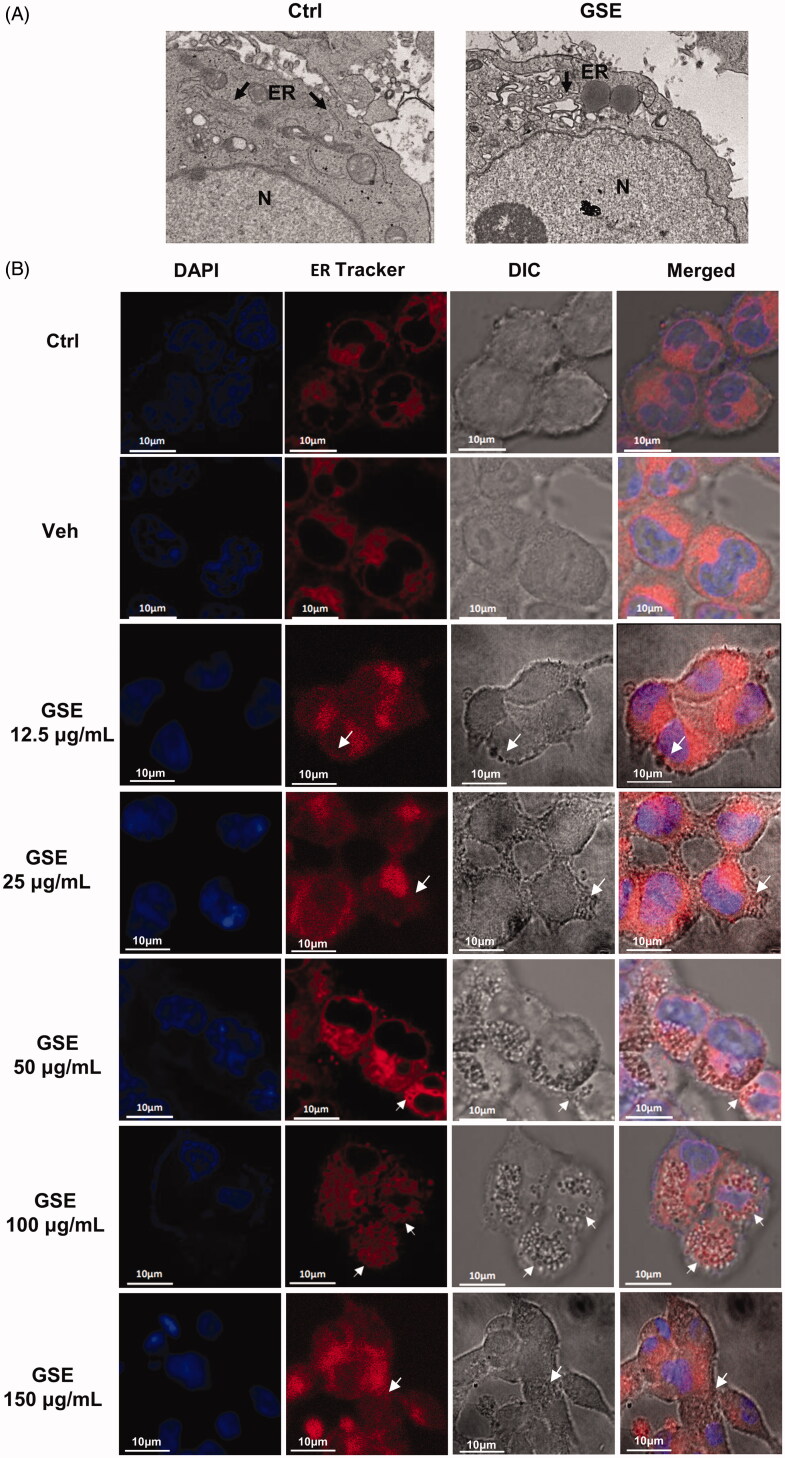
GSE induces cytoplasmic vacuolation originating from the endoplasmic reticulum (ER) in HepG2 cells. (A) Swelling of the ER in HepG2 cells after treatment with 100 μg/mL of GSE for 24 h. Cells were visualized via transmission electron microscopy. Arrowheads indicate the ER. (B) Cytoplasmic vacuolation in HepG2 cells resulting from enlargement of the ER. HepG2 cells were incubated with ER-Tracker Red dye (1 μM) after exposure to GSE for 24 h and were observed via a fluorescence microscope (scale bars, 10 μm). Ctrl: control; Veh: vehicle; N: nuclear: ER: endoplasmic reticulum; DIC: differential interference contrast.

To confirm the origination of the GSE-induced vacuoles, we used TEM to visualize the fine structure of HepG2 cells. We observed extensive swelling of the ER with intact nuclei in GSE-treated cells (Figure 3(A)). The vacuoles appeared clear and did not contain cytoplasmic material. We further investigated the relationship between the cytoplasmic vacuolization and the ER by staining with ER-Tracker Red. We observed the appearance of vacuoles after exposure to GSE, and the vacuolar membranes were positive for an ER-specific marker (Figure 3(B)). These findings suggest the cytoplasmic vacuolation induced by GSE in HepG2 cells is primarily due to extensive dilation of the ER.

### GSE induces ER stress and activates the UPR

**Figure 4.**
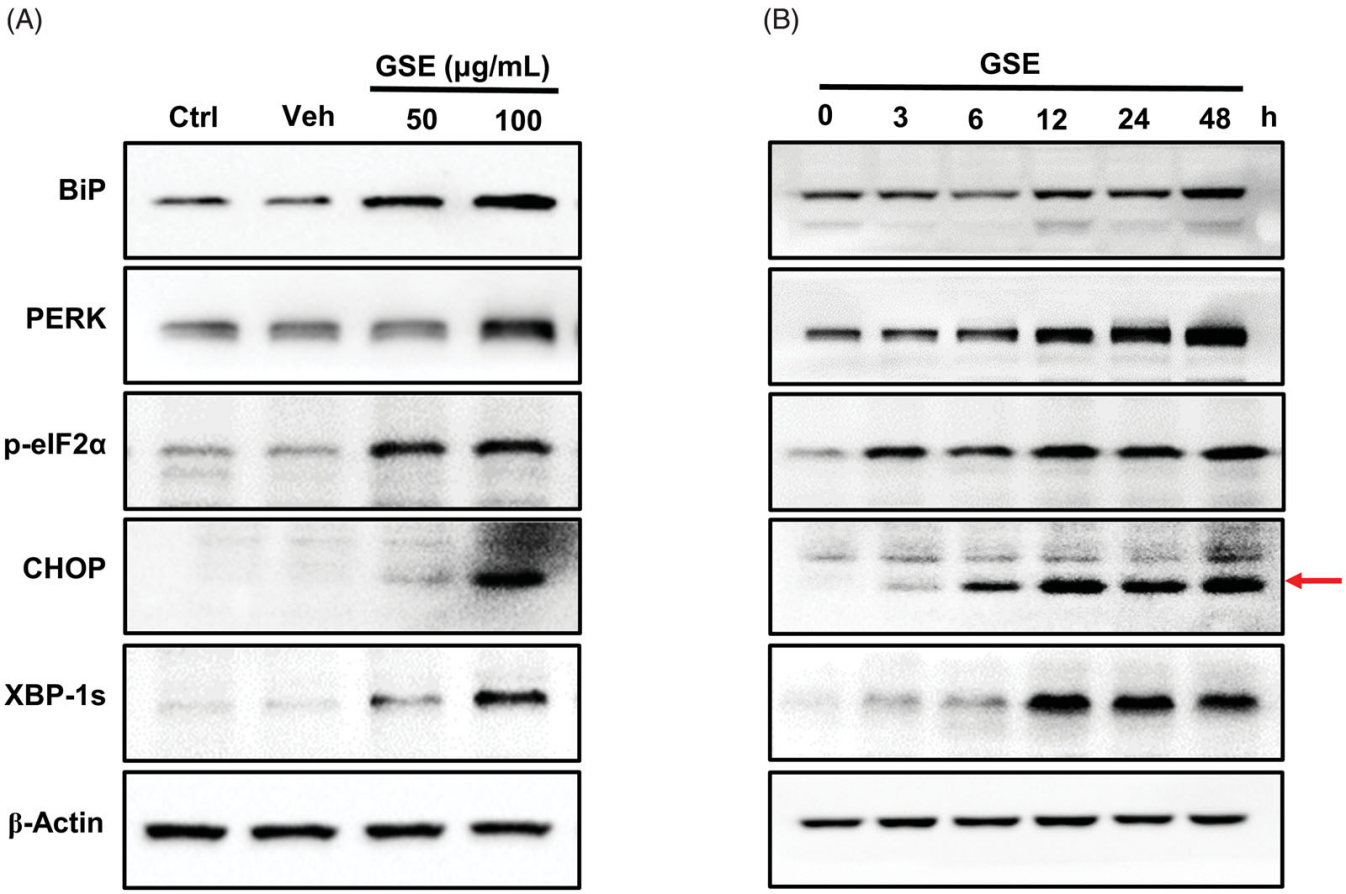
GSE induces endoplasmic reticulum stress and activates the unfolded protein response pathway in HepG2 cells. The expression of ER stress-associated proteins in HepG2 cells after GSE treatment. (A) Cells were treated with GSE at indicated concentration for 24 h. (B) Cells were treated with 100 μg/mL of GSE with difference time exposure. β-Actin was used as a loading control in the western blot analysis. Ctrl: control; Veh: vehicle; p: phosphorylated.

ER vacuolization is usually associated with persistent ER stress. When ER stress occurs, the immunoglobulin heavy-chain binding protein or binding-immunoglobulin protein (BiP), a chaperone protein combined with unfolded or misfolded proteins to promote the correct folding of newborn proteins, activates the UPR signalling pathway (Gardner et al. 2013). We found BiP expression was significantly increased in GSE-treated HepG2 cells at 12, 24 and 48 h exposure via western blotting. Furthermore, GSE promoted BiP expression gradually with the increase of GSE concentration (Figure 4(A)).

One branch of the UPR signalling pathway is mediated by PERK. PERK undergoes oligomerization and phosphorylates the eukaryotic translation initiation factor 2α-subunit (eIF2α), thereby inhibiting protein translation (Clarke et al. 2014). We found GSE increased levels of PERK and phosphorylated eIF2α in HepG2 cells (Figure 4). As well as inhibiting protein translation, phosphorylated eIF2α promotes the expression of the transcription factor C/EBP homologous protein (CHOP). In parallel with the induction of eIF2α phosphorylation, GSE treatment also upregulated CHOP expression in HepG2 cells (Figure 4).

When the UPR is activated, IRE1 oligomerizes and activates its ribonuclease domain, which catalyses the splicing of a ubiquitously expressed form of the X-box binding protein 1 (XBP-1u) to generate an isoform named XBP-1s. Therefore, XBP-1s serves as a marker of IRE1 activation (Nishitoh et al. 2002). We found GSE treatment increased XBP-1s protein levels in HepG2 cells (Figure 4). Together, these results demonstrate GSE induces the ER stress-activated UPR pathway in HepG2 cells.

### GSE arrests the cell cycle in the G2/M phase

**Figure 5.**
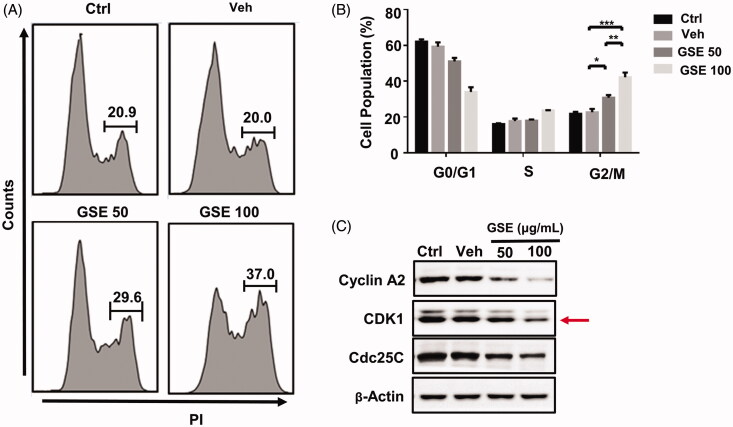
GSE arrests HepG2 cells at the G2/M stage and affects expression of cell cycle regulators. (A) Cell cycle progression of HepG2 cells after treatment with GSE over a period of 24 h; and quantitative distribution of HepG2 cells in different phases of the cell cycle. (B) Data are shown as mean values ± SEM (*n* = 3). Statistical analysis was carried out by a one-way analysis of variance (ANOVA) followed by Tukey’s pairwise comparisons and *p* < 0.05 was considered statistically significant. *p < 0.05, **p < 0.01 and ***p < 0.001. (C). Western blot analysis of cell cycle regulatory proteins in HepG2 cells after treatment with GSE for 24 h. Ctrl: control; Veh: vehicle; GSE 50: GSE 50 μg/mL; GSE 100: GSE 100 μg/mL.

Next, we examined whether GSE inhibits the growth of HepG2 cells via cell cycle arrest using flow cytometry. Compared to the control group, GSE treatment increased the proportion of cells in the G2/M phase with the increase of GSE concentration (30.8 ± 1.4% and 42.2 ± 2.6% at a GSE concentration of 50 and 100 μg/mL, respectively; vs. 21.03 ± 1.10%, control, Figure 5(A,B)). In addition, the proportion of cells in the G0/G1 phase was reduced, yet no changes were observed in the S phase (Figure 5(A,B)).

Cell-cycle progression requires the activity of regulatory cyclins and their catalytic partners, the cyclin-dependent kinases (CDKs). Using western blot analysis, we found GSE treatment decreased the protein levels of cyclin A2, cyclin-dependent kinase 1 (CDK1) and Cdc25C in HepG2 cells (Figure 5(C)). These results indicate the inhibition of cell proliferation by GSE is partly associated with the induction of G2/M phase arrest in HepG2 cells.

### ER stress is involved in GSE-induced G2/M phase cell cycle arrest

**Figure 6.**
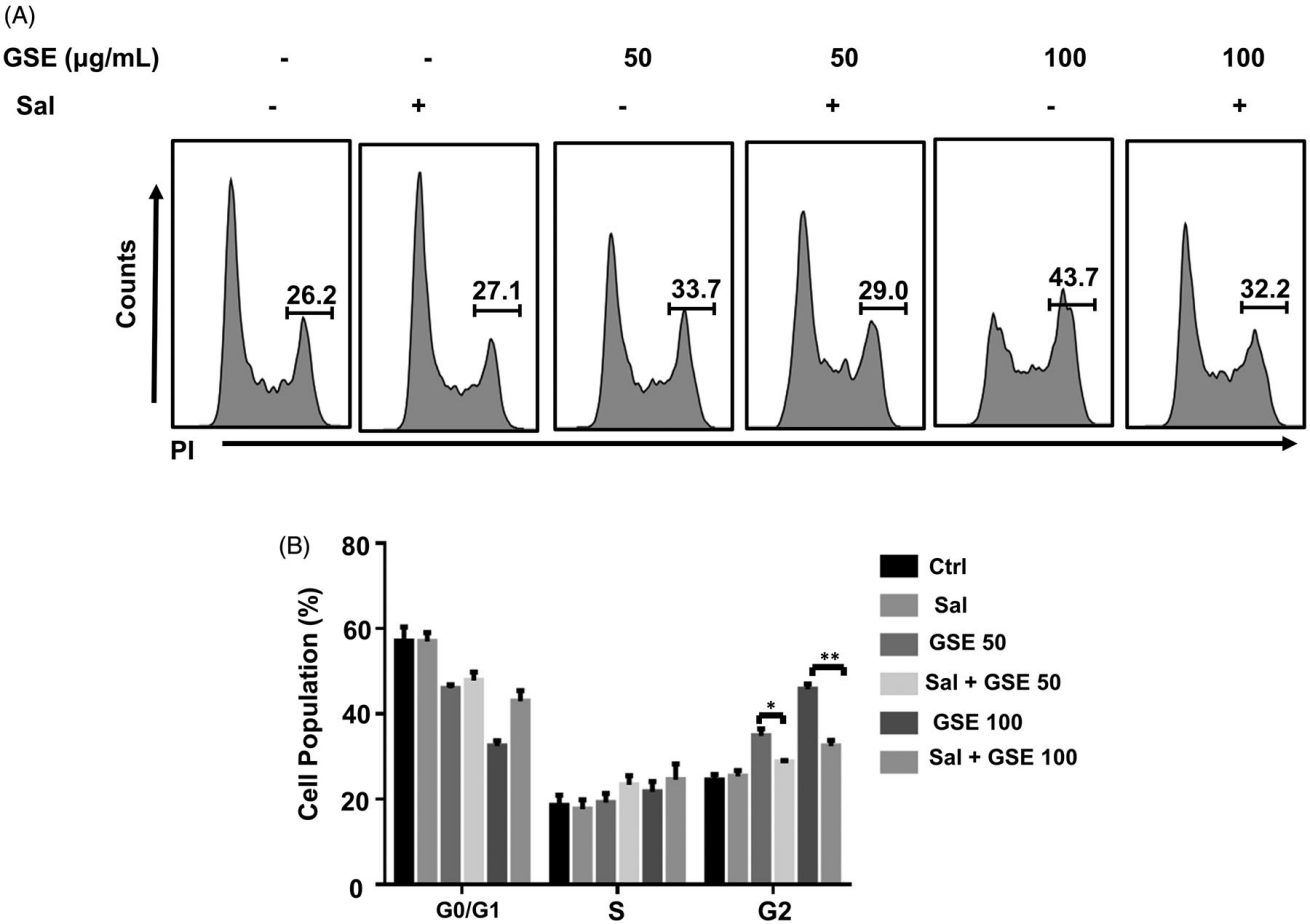
Salubrinal attenuates GSE-induced G2/M cell cycle arrest. (A) HepG2 cells were pre-treated with 25 mM salubrinal for 1 h and further treated with GSE for 24 h to examine cell cycle progression by flow cytometry. (B) Data are shown as mean values ± SEM (*n* = 3). Statistical analysis was carried out by a one-way analysis of variance (ANOVA) followed by Tukey’s pairwise comparisons and *p* < 0.05 was considered statistically significant. *p < 0.05 and **p < 0.01. Ctrl: control: Sal: salubrinal; GSE 50: GSE 50 μg/mL; GSE 100: GSE 100 μg/mL.

Since recent studies have suggested that UPR activation affects cell cycle protein expression and induces cell cycle arrest in dividing cells (Brewer et al. 1999), we investigated whether the ER stress induced by GSE regulates the cell program in HepG2 cells. Salubrinal, a chemical inhibitor of ER stress, selectively attenuates the dephosphorylation of p-eIF2a and has been reported to alleviate cells from ER stress (Boyce et al. 2005). We found cells co-treated with salubrinal and GSE significantly decreased the proportion of HepG2 cells in the G2/M phase (29.1 ± 1.1% and 32.2 ± 1.3% at a GSE concentration of 50 and 100 μg/mL, respectively) compared to GSE treatment alone (33.7 ± 1.0%, *p* < 0.05 and 43.7 ± 1.2%, *p* < 0.01; Figure 6). These findings suggest ER stress is involved in GSE-induced G2/M phase cell cycle arrest.

### GSE has a negligible effect on the apoptosis

**Figure 7.**
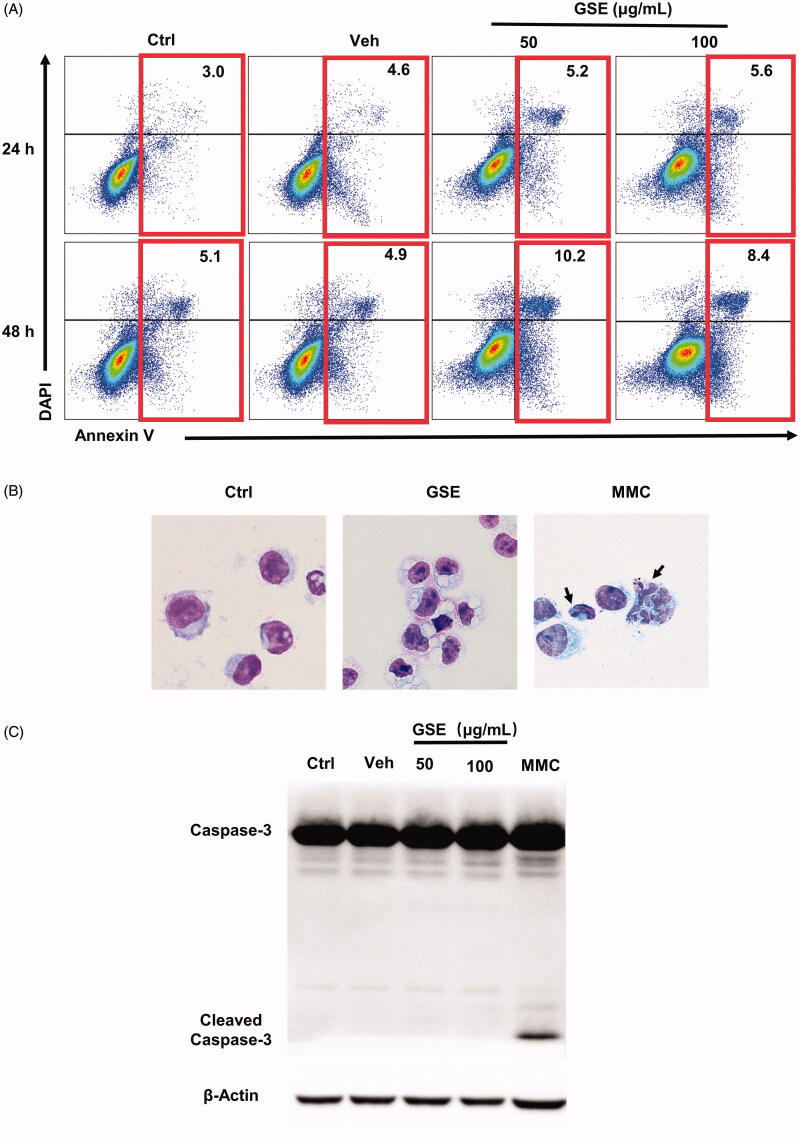
GSE has a negligible effect on the apoptosis of HepG2 cells. (A) Analysis of HepG2 cell apoptosis following GSE treatment. HepG2 tumour cells were cultured with GSE at indicated doses or with emulsion (vehicle), harvested and stained with Annexin V-FITC and DAPI. Numbers in the flow cytometric plots indicate the proportions of gated cells. (B) Cells were stained with Wright–Giemsa staining after treatment with 100 μg/mL GSE for 24 h. Cells were treated with MMC (50 μg/mL) for 24 h as a positive control (original magnification, ×400). Arrowhead indicates a typical fragmented nucleus of a cell undergoing apoptosis. (C) Western blot showing protein levels of caspase-3, cleaved-caspase-3. Ctrl: control: Veh: vehicle: MMC: mitomycin.

It has been reported that apoptosis of cancer cells was induced by *Ganoderma* extract (Wu et al. 2013). However, flow cytometry using FITC-conjugated Annexin V revealed that GSE exposure did not induce apoptosis of cancer cells (Figure 7(A)). Meanwhile, GSE treatment did not change the expression of apoptosis-related protein, cleaved-caspase-3 (Figure 7(C)). In addition, GSE treatment did not cause nucleic condensation or fragmentation in HepG2 cells (Figure 7(B)). These findings suggest that GSE did not generate apoptosis of HepG2 cells.

## Discussion

Although *Ganoderma* extract has been extensively reported to show antitumor activity against a variety of human cancers, including lung, colon, bladder, cervical, liver and breast cancer (Hu et al. 2002; Lin et al. 2003; Lu et al. 2004; Yang 2005; Wu et al. 2013; Oliveira et al. 2014; Liu et al. 2015, 2020; Ruan et al. 2015; Hsin et al. 2016; Chan et al. 2017; Li et al. 2017; Jiao et al. 2020), its mechanism of action requires further investigation. This study provides evidence that an extract from *G. sinensis* spores effectively inhibits proliferation and induces G2/M cell arrest in HepG2 cells. We also found GSE induces ER stress and activates the UPR in HepG2 cells. Furthermore, we showed GSE suppressed the proliferation of HepG2 cells by arresting the cell cycle in the G2/M phase partly through inducing ER stress. Furthering the understanding of the antitumor mechanisms of *G. sinensis* will allow researchers and clinicians to utilize this drug for cancer treatment better.

*Ganoderma lucidum* (Leyss. ex Fr.) Karst. and *G. sinensis* are recorded by Chinese Pharmacopoeia (2015Ed) as among the more than 90 *Ganoderma* strains that are indigenous to China. To date, most research has focussed on *G. lucidum*. For example, accumulating evidence shows the effects of *G. lucidum* triterpenoid-enriched extracts (GLETs) on proliferation inhibition are directly related to their ability to induce cell cycle arrest (Wu et al. 2013). Interestingly, the phase of cell cycle arrest of GLETs vary according to the cell line; for example, GLETs induce cell cycle arrest at the G1 phase in breast, lung and colon cancer cells (Lin et al. 2003), while they induce G2/M arrest in hepatoma cells (Lin et al. 2003; Wu et al. 2013). Similarly, we found treatment with GSE (triterpene-enriched extracts) inhibited G2/M progression in HCC cancer cells. Cell cycle progression is regulated by cell division cyclins and CDKs, and G2 arrest could be due to a deficiency in M phase promoting factors (Molinari 2000). Indeed, our data showed that cyclin A2, CDK1 and Cdc25C were downregulated by GSE treatment in HepG2 cells, which is consistent with G2/M phase arrest.

Triterpenoid bioactive compounds extracted from *G. lucidum* have also been shown to cause stress in HCC cancer cells (Lin et al. 2003). Recently, it was reported that the UPR activated by ER stress affects cell cycle protein expression and induces a cell cycle arrest in dividing cells (Brewer et al. 1999; Kim et al. 2008). In the present study, we found GSE treatment resulted in the increased expression of various ER stress markers, including BiP, PERK, CHOP, XBP1s and p-eIF2α. Using electron microscopy and ER-Tracker Red staining, we observed GSE treatment significantly induced ER dilatation and vacuolization in HepG2 cells. Based on these findings, we hypothesized salubrinal, which inhibits global protein translation by blocking the dephosphorylation of p-eIF2a, may reduce the protein burden of the ER and protect cells from ER stress, thus promoting cell cycle progression. We found salubrinal treatment significantly attenuated GSE-induced G2/M cell cycle arrest, which suggests GSE induces G2/M phase cell cycle arrest in HepG2 cells, at least in part, through causing ER stress.

The folding ability of the ER could be disturbed by various endogenous and exogenous insults that result in ER stress (Kim et al. 2008). When the pressure is excessive or lasts too long, ER stress turns into a toxic signal, leading to apoptosis. In the present study, no alteration in the sub-G1 peak was observed in the cell cycle analysis of HepG2 cells following treatment with GSE, indicating apoptosis was not involved (Figure 5). Furthermore, the analysis of morphology and Wright–Giemsa staining or Annexin V/DAPI assay also showed no occurrence of apoptosis (Figure 7(A,B)). Similarly, the expression of cleaved caspase‑3 was not detected in HepG2 cells via western blot, suggesting GSE does not induce apoptosis (Figure 7(C)). Consistent with these results, Lin et al. (2003) found that triterpene-enriched extracts from *G. lucidum* caused G2 phase cell cycle arrest in HCC cancer cells without inducing apoptosis. Indeed, recent studies have revealed a novel mode of non-apoptotic programmed cell death, termed paraptosis-like cell death, which is characterized by extensive cytoplasmic vacuolation due to dilation of the ER and mitochondria and lacks the typical apoptotic hallmarks (Sperandio et al. 2000; Yumnam et al. 2016; Xue et al. 2018). However, whether GSE-induced ER stress results in paraptosis-like cell death needs further investigation.

## Conclusions

This work describes the anti-proliferative effects of GSE in HCC cancer cell, and details its underlying mechanism of action by inducing cell cycle arrest. We propose a new mechanism of action of GSE against HCC by inducing G2/M phase cell cycle arrest through activating the ER stress pathway. These results provide insight into some potential therapeutic strategies for HCC.
